# Advances in neurexin studies and the emerging role of neurexin-2 in autism spectrum disorder

**DOI:** 10.3389/fnmol.2023.1125087

**Published:** 2023-02-27

**Authors:** Sheraz Khoja, Mulatwa T. Haile, Lulu Y. Chen

**Affiliations:** Department of Anatomy and Neurobiology, School of Medicine, University of California, Irvine, Irvine, CA, United States

**Keywords:** synapses, autism spectrum disorder, synaptic signaling, neurexins, excitatory/inhibitory balance, synaptopathy, genetics, social behavior

## Abstract

Over the past 3 decades, the prevalence of autism spectrum disorder (ASD) has increased globally from 20 to 28 million cases making ASD the fastest-growing developmental disability in the world. Neurexins are a family of presynaptic cell adhesion molecules that have been increasingly implicated in ASD, as evidenced by genetic mutations in the clinical population. Neurexins function as context-dependent specifiers of synapse properties and critical modulators in maintaining the balance between excitatory and inhibitory transmission (E/I balance). Disrupted E/I balance has long been established as a hallmark of ASD making neurexins excellent starting points for understanding the etiology of ASD. Herein we review neurexin mutations that have been discovered in ASD patients. Further, we discuss distinct synaptic mechanisms underlying the aberrant neurotransmission and behavioral deficits observed in different neurexin mouse models, with focus on recent discoveries from the previously overlooked neurexin-2 gene (*Nrxn2* in mice and *NRXN2* in humans). Hence, the aim of this review is to provide a summary of new synaptic insights into the molecular underpinnings of ASD.

## 1. Introduction

Autism spectrum disorder (ASD) is characterized by two core criteria according to the Diagnostic and Statistical Manual of Mental Disorders criteria: (1) impaired social communication and interaction, and (2) restricted and repetitive interests and behaviors (Landa, 2008; Lai et al., 2014; Rylaarsdam and Guemez-Gamboa, 2019). ASD is the leading cause of disability for children under 5 in the world (Baxter et al., 2015). The Autism and Developmental Disabilities Monitoring (ADDM) Network reported a significant increase in prevalence of ASDs among children from 1 in 150 in 2000–2002 to 1 in 68 in 2010–2012 (Autism and Developmental Disabilities Monitoring Network Surveillance Year 2008 Principal Investigators and Centers for Disease Control and Prevention, 2012; ADDM and CDC, 2014; Christensen et al., 2016) and then to 1 in 44 in 2018 (Maenner et al., 2021) in the US, emphasizing the importance of increased scientific research. The prevalence of ASD is more common in males over females with a ratio of 4:1 (Christensen et al., 2016; Baio et al., 2018). ASD require life-long support of special educational and social services with an estimated cost of $461 billion by 2025 (Lavelle et al., 2014; Leigh and Du, 2015), thus imposing a major societal and economic burden in the US. Over 70% of individuals with ASD have concurrent conditions; among these 45% have intellectual disability, 14–38% have tic disorders, 8–30% have epilepsy, 5% have genetic syndromes (i.e., Fragile X, Rett syndrome, Angelman syndrome etc.), 50–80% have sleep disorders, 42–56% have anxiety, 12–70% have depression, and up to 32% have various personality disorders (Lai et al., 2014). Given that ASD is a multifactorial and complex disorder, it is currently believed that it occurs due to a combination of genetic and environmental risks that either directly or indirectly affect synapses (Persico and Bourgeron, 2006; Spooren et al., 2012; Bourgeron, 2015; Masini et al., 2020). The rising prevalence, economic and social hardships, and co-morbidity with other neurological disorders emphasizes the need to better understand the pathophysiology at the genetic and cellular level, in order to discover drug targets and develop effective therapeutic interventions.

Genetic causes have been reported to contribute to 25% of ASD cases (Huguet et al., 2013). Studies have attributed a strong genetic component to the pathophysiology of ASD as evidenced by a high concordance rate of 82–90% in monozygotic twins, compared with 1–10% in dizygotic twins and 2–3% in siblings (Folstein and Rosen-Sheidley, 2001; Veenstra-Vander Weele and Cook, 2004). 10–25% of ASD cases have an underlying genetic disorder such as Fragile X, tuberous sclerosis complex, or Rett syndrome (Carter and Scherer, 2013). Genome-wide association studies and next generation sequencing have made significant breakthroughs in our understanding of ASD by identifying short insertions or deletions, copy number variants (CNVs), single nucleotide variants (SNVs) and single nucleotide polymorphisms (SNPs). CNVs have been reported to occur at a rare frequency in ASD with 3% of ASD cases associated with recurrent CNVs (Bourgeron, 2016) and 4–10% of ASD cases with *de novo* CNVs (Sebat et al., 2007; Pinto et al., 2010). SNVs have been found to occur in 7% of ASD cases (Masini et al., 2020).

Several of the ASD-candidate genes, identified through gene testing techniques, have a critical role in synapse assembly, transmission, and plasticity. To this end, numerous investigations have identified genetic mutations in synaptic cell adhesion molecules (SAMs) responsible for mediating trans-synaptic signaling, shaping synapse properties, and defining the characteristics of neural networks (Chih et al., 2005; de Wit et al., 2009; Siddiqui et al., 2010; Aoto et al., 2013; Pettem et al., 2013; Anderson et al., 2015; Aoto et al., 2015; Luo et al., 2021). SAMs are integral to organizing synaptic junctions, mediating synaptic transmission and plasticity (Missler et al., 2012; Jang et al., 2017; Südhof, 2018; Kim et al., 2021; Südhof, 2021). Synapses are the most fundamental structures that connect neurons into circuits by enabling the transfer of information, while simultaneously processing this information during transfer; thus governing cognition and behavior (Williams et al., 2010; Bargmann, 2012; Bargmann and Marder, 2013; Di Cristo and Chattopadhyaya, 2020; Sanes and Zipursky, 2020). Normal brain development is accompanied by dynamic synaptic changes which regulate a balance of excitatory glutamatergic and inhibitory GABAergic neuronal firing (E/I balance). E/I imbalances in key cortical and subcortical neural circuits have been proposed as an etiological mechanism underlying ASD (Rubenstein and Merzenich, 2003; Oliveira et al., 2018; Port et al., 2019; Culotta and Penzes, 2020). To this end, numerous studies have reported altered levels of glutamate (Shinohe et al., 2006; Carlson, 2012; He et al., 2021) and/or GABA levels in ASD patients (Fatemi et al., 2010; Robertson et al., 2016; Schür et al., 2016). Considering their critical role in neurotransmission, SAMs are key players involved in excitatory and inhibitory synapse assembly and transmission and hence, are integral to modulating the E/I balance. The discovery of novel genetic mutations in SAMs plays an essential role in elucidating of specific pathways and mechanisms associated with ASD endophenotypes, which can aid in the development of druggable targets at synapses. These investigations are important considering the heterogeneity in the severity and magnitude of core ASD phenotypes, which makes diagnoses challenging and limits pharmacotherapeutic options.

Neurexins are multifaceted representatives of presynaptic SAMs that interact with a wide variety of postsynaptic partners (neuroligins, dystroglycans, cerebellins, and LRRTMs) in mediating diverse synaptic functions including synapse assembly (Graf et al., 2004; Nam and Chen, 2005; Kang et al., 2008), presynaptic release machinery (Dean et al., 2003; Anderson et al., 2015; Luo et al., 2020), postsynaptic receptor signaling, and synaptic function (Aoto et al., 2013, 2015; Chen et al., 2017; Luo et al., 2021). The mammalian genome contains three neurexin genes (*Nrxn1, Nrxn2, Nrxn3* in mice; and *NRXN1, NRXN2, NRXN3* in humans), and each gene contains two different promoters initiating neurexin gene transcription from two different sites referred to as α-neurexins with longer coding sequences and therefore, larger membrane-tethered moieties, and β-neurexins with shorter sequences and consequently smaller membrane-tethered moieties (Ushkaryov et al., 1992; Ushkaryov and Südhof, 1993; Ushkaryov et al., 1994). Neurexin-1 gene has an additional promoter which initiates neurexin gene transcription of γ-neurexin with the shortest sequence and smallest membrane-tethered moiety. The extracellular sequence of Nrxn1γ contains only the juxtamembranous threonine rich residues and cysteine-loop sequences of Nrxn1α and Nrxn1β but not the typical extracellular neurexin sequences (Sterky et al., 2017). An intriguing feature of neurexins is that they can undergo extensive alternative splicing at six canonical sites (referred to as SS1-SS6), leading to generation of thousands of splice variants, each of which is capable of conferring specific synaptic properties (Ullrich et al., 1995; Missler and Südhof, 1998; Tabuchi and Südhof, 2002). Neurexins have been exceedingly implicated in neuropsychiatric disorders, especially ASD and its comorbidities. In humans each of the three neurexin genes have been implicated in ASD [*NRXN1* (Kim et al., 2008; Marshall et al., 2008; Glessner et al., 2009; Onay et al., 2016; Ishizuka et al., 2020); *NRXN2* (Gauthier et al., 2011; Mohrmann et al., 2011; Wang et al., 2018); *NRXN3* (Vaags et al., 2012; Wang et al., 2018; Yuan et al., 2018)]. At the preclinical level, mouse models have shown that deletion of neurexin genes induced behaviors reminiscent of ASD phenotypes [*Nrxn1* (Etherton et al., 2009; Grayton et al., 2013; Rabaneda et al., 2014; Armstrong et al., 2020); *Nrxn2* (Dachtler et al., 2014; Born et al., 2015; Dachtler et al., 2015; Haile et al., 2022)]. Understanding the role of neurexins in synaptic signaling will substantially improve ASD prognosis. The present review focuses on current findings in neurexins that have advanced our understanding of ASD, with particular emphasis on *NRXN2* as an emerging ASD risk gene candidate.

## 2. Neurexin mutations associated with human ASD patients

According to the ClinVar database, provided by the National Center for Biotechnology Information for collecting genotype–phenotype relationships in the human genome, over 19,000 patients with ASD have one or more genetic mutations in over 18,000 different genes (ClinVar, Supplementary Data). This is especially important because recent work showed that there are 20,000 protein coding genes in the human genome suggesting the increased risk for ASD in the human population (Nurk et al., 2022). The abundance of gene mutations found in patients with ASD may contribute to the diverse variation in the clinical manifestation of ASD. Among these, 0.08% of total reports are those in presynaptic neurexins alone, and 0.16% in either neurexins or neuroligins. Given the considerable number of mutations linking neurexins to ASD, longitudinal studies are required to fully understand how these mutations may lead to a predisposition to ASD.

### 2.1. NRXN1 and NRXN3

To date 1,756 mutations in the *NRXN1* gene have been reported in humans. Among these, 0.5% are in patients with ASD. Several studies (Kim et al., 2008; Marshall et al., 2008; Zahir et al., 2008; Glessner et al., 2009; Onay et al., 2016; Ishizuka et al., 2020) provide evidence that single point mutations and CNVs in *NRXN1,* found in human patients with ASD, can lead to pleiotropic effects. Fewer studies have investigated *NRXN3* mutations in human ASD patients (Vaags et al., 2012; Wang et al., 2018; Yuan et al., 2018). According to ClinVar, 39 mutations in *NRXN3* have been reported in patients, and 10% are in patients with ASD.

### 2.2. NRXN2

*NRXN2* gene was not implicated in ASD in the literature or any disorder until 2011, almost 20 years from the initial discovery of the gene (Gauthier et al., 2011; Mohrmann et al., 2011). Gauthier et al. identified a truncating mutation in exon 12 of *NRXN2* in a patient with ASD, who inherited this mutation from a father with severe language delay and a family history of schizophrenia. Gauthier et al. engineered this mutation and transfected it into COS cells. They discovered that this mutation was causing a failure of Nrxn2 to anchor into the plasma membrane and was unable to bind postsynaptic binding partners Lrrtm2 or Nlgn2 in cell binding assays. Since then, other studies have observed *de novo* deletions in *NRXN2* gene in human patients with ASD, including one missing a chromosomal region spanning the entire *NRXN2* gene that was suspected to be associated with the patient’s autistic traits and neurodevelopmental delay (Mohrmann et al., 2011), the other was identified in a patient exhibiting autistic behavior and severe intellectual disability with a 1.6 Mb deletion at the *NRXN2* locus (Boyle et al., 2015). Other reports have also identified *NRXN2* mutations in human patients, including a more recent study of a patient containing heterozygous variants in *NRXN1* and *NRXN2* genes, with early infantile epileptic encephalopathy (EIEE; Rochtus et al., 2019). This patient inherited the *NRXN2* mutation from a father with a family history of febrile seizures. Currently, according to ClinVar, a total of 120 mutations have been identified, and among those, 5% have ASD, 5% have intellectual disability, and 2.5% have epilepsy (Supplementary Data).

Mutations in all three neurexin genes have been found in disorders comorbid with autism such as epilepsy (Tuchman et al., 2010; Jeste and Tuchman, 2015), indicating that neurexin mutations may represent a common genetic mechanism underlying autism-epilepsy co-morbidity. Thus, investigating the synaptic significance of neurexins in circuitries regulating behavioral functions (cognition, information processing, and emotional responses) can be a stepping stone towards unraveling the etiology of these disorders and developing therapeutic intervention.

## 3. Neurexin mediated regulation of synaptic density and transmission

### 3.1. Nrxn1αβ, Nrxn2αβ, and Nrxn3αβ

Since their initial discovery as type I membrane receptors for α-latrotoxin, a spider toxin known to induce excessive synaptic vesicle exocytosis resulting in neuromuscular paralysis (Ushkaryov et al., 1992; Ushkaryov and Südhof, 1993; Ushkaryov et al., 1994), several studies have shown that neurexins play critical roles in diverse synaptic functions including synapse assembly (Missler et al., 2003; Nam and Chen, 2005; Luo et al., 2020), presynaptic release machinery (Missler et al., 2003; Anderson et al., 2015; Aoto et al., 2015; Chen et al., 2017; Luo et al., 2020, 2021), and postsynaptic receptor signaling (Missler et al., 2003; Aoto et al., 2013, 2015; Chen et al., 2017).

Initial studies using constitutive deletion approach provided the first evidence that α-neurexins are required for postnatal survival. Homozygous deletion of Nrxn1/2/3 (only α-neurexins) reduced presynaptic Ca^2+^ influx, number of GABAergic terminals, and reduced spontaneous and evoked neurotransmitter release in neocortical neurons (Missler et al., 2003) and at the neuromuscular junctions (Sons et al., 2006). Further studies using homozygous Nrxn1/2/3α knockout (KO) mouse model showed that they regulate N and P/Q type Ca^2+^ channels (Zhang et al., 2005) and are required for normal NMDA receptors (NMDARs) function (Kattenstroth et al., 2004). The role of neurexins in regulating neurotransmission through coupling of presynaptic calcium channels to release sites was later discovered by using a conditional KO (cKO) approach deleting Nrxn1/2/3 (all α-and β-neurexins) (referred to as “pan-Nrxn cKO” approach) at the Calyx of Held synapses (Luo et al., 2020). Our understanding of neurexins was greatly enhanced when the conditional KO approach became available and allowed for postnatal survival with both spatial and temporal controls. Studies using a cKO approach of Nrxn1/2/3 (only β-neurexins) showed that they regulate postsynaptic endocannabinoid signaling through suppressing CB1 receptor agonist 2AG synthesis in hippocampal synapses (Anderson et al., 2015). Interestingly, despite the low expression of β-neurexins (10- to 100-fold lower than that of α-neurexins), cKO of Nrxn1/2/3 (only β-neurexins) lead to a two-fold decrease in three excitatory synapse parameters: (1) EPSC amplitude, (2) release probability, and (3) action potential induced Ca^2+^ influx (Anderson et al., 2015).

Recent work has reported that neurexins are required for presynaptic GABA_B_ receptor signaling (Luo et al., 2021). The study employed a pan-Nrxn cKO approach in four central synapses: excitatory Calyx of Held synapses in the brainstem, excitatory and inhibitory synapses in pyramidal neurons in the CA1 region of the hippocampus, and inhibitory basket cell synapses in the cerebellum, thus showing that neurexins universally regulate presynaptic GABA_B_-receptor signaling, albeit at different magnitude between synapses (Luo et al., 2021). More recent work shows that neurexins regulate GABA co-release in dopamine (DA) neurons (Ducrot et al., 2021). The pan-Nrxn cKO approach in DA neurons, showed (1) region-specific increase in GABA release from DA terminals (increase in the ventral but not in the dorsal striatum) and (2) slower DA reuptake along with decreased and increased densities of DA transporter (DAT) and vesicular monoamine transporter (VMAT2), respectively (Ducrot et al., 2021), indicating that neurexins support the functional but not the structural aspect of DA neuron synapses. Utilization of the conditional deletion approach has enabled significant progress in our understanding of neurexins’ function in specific synapses (Ducrot et al., 2021; Luo et al., 2021). This is important because the first study using the pan-Nrxn cKO approach in various types of synapses discovered severe, but dramatically different phenotypes (Chen et al., 2017). This study showed that ablation of neurexins from parvalbumin-positive interneurons in the prefrontal cortex caused a loss of synapses without any effect on action-potential induced Ca^2+^ influx, or synaptic strength. The same study revealed that ablation of neurexins from somatostatin-positive interneurons in the same brain region impaired action-potential induced Ca^2+^ influx and decreased synaptic strength without changes in number of synapses (Chen et al., 2017). This shows that conditional deletion of neurexins induced two different synaptic phenotypes in two inhibitory presynaptic neurons that target the same postsynaptic neuron. Taken together, these studies indicate that neurexins promote the functional properties, but not the physical assembly and organization of synapses. Additionally, these findings suggest that neurexins do not perform canonical functions at the synapses that are generalized, universal functions across all synapses. Instead, neurexins can perform distinct functions in different synapses and hence, they should be investigated in a cell-type and circuit-specific manner (Table 1).

**Table 1:** Summary table of synaptic transmission examined in neurexin mutant mouse models.

Neurexin targeted in mouse models	Brain region examined	Genetic approach/manipulation	Main findings in synaptic function	References
Nrxn1α, β, or αβ	Cortex^1^, Hippocampus^2,3^	Conditional KO^1^	Nrxn1β KO decreased mEPSCs and mIPSCs frequency in cortical layer 5/6 pyramidal neurons^1^	Rabaneda et al.(2014)^1^
		Constitutive KO^2^	Nrxn1α KO reduced spontaneous release in excitatory glutamatergic synapses without any effect on inhibitory transmission in pyramidal neurons in the CA1 region of the hippocampus^2^	Etherton et al. (2009)^2^
		Conditional KI^3^	Conditional KI of SS4+ in Nrxn1 increased NMDARs-mediated EPSCs without affecting AMPARs-mediated EPSCs in the CA1-subiculum synapses in the hippocampus^3^	Dai et al. (2019)^3^
Nrxn2α, β, or αβ	Hippocampus^3,5,6^	Conditional KI^3^	Conditional KI of SS4+ in Nrxn2 lead to no change in NMDAR and AMPAR-mediated EPSCs^3^	Dai et al. (2019)^3^
	Cortex^4^	Constitutive KO^4,5^	Constitutive KO of Nrxn2α and Nrxn2αβ reduced spontaneous transmitter release at excitatory synapses in the neocortex. Both KO mice exhibited altered facilitation and NMDAR function due to a reduction in NMDAR-dependent decay time and responses in excitatory synapses. Inhibitory transmission and synapse densities and ultrastructure remained unchanged in both Nrxn2α and Nrxn2αβ KO^4^	Born et al. (2015)^4^
		Conditional KO^5,6^	Constitutive deletion of *Nrxn2* gene increases hippocampal CA3 to CA1 synaptic connections. Conditional (Neuron-specific) deletion of *Nrxn2* gene also increases CA3 to CA1 synaptic connectivity, and release probability, and increases excitatory synapse density in the CA1 region^5^	Lin et al. (2023)^5^
			Conditional (Emx1Cre driven) deletion of *Nrxn2* gene increased network activity in hippocampal circuities as measured by increased sEPSC and sIPSC frequencies independent of changes in neurotransmitter release probability or changes in AMPARs and NMDARs contributions^6^	Haile et al. (2022)^6^
Nrxn3α, β, or αβ	Hippocampus^3,7^	Conditional KI^3^	Conditional KI of SS4+ in Nrxn3 suppressed AMPARs-mediated synaptic responses without any effect on NMDARs function in the same synapses^3^	Dai et al. (2019)^3^
		Constitutive KI^7^	Constitutive KI of SS4+ in Nrxn3 caused a decrease in AMPARs mediated synaptic responses in hippocampal synapses^7^	Aoto et al. (2013)^7^
	Olfactory bulb^8^	Conditional KO^8^	Conditional deletion of *Nrxn3* gene in the CA1 region of the hippocampus decreased (AMPARs)-mediated excitatory response and blockade of NMDARs-mediated LTP. In olfactory bulb synapses however, conditional deletion of *Nrxn3* gene decreased GABAergic mediated inhibitory responses^8^	Aoto et al. (2015)^8^
Nrxn123α, β, or αβ	Cortex^9,11^, Hippocampus^12,14^ Brain Stem^9,10,13,14^ Cerebellum ^11,14^ Striatum^15^	Constitutive KO^9,10^	Constitutive deletion of Nrxn1/2/3 (only α-neurexins) reduced presynaptic Ca^2+^ influx, number of GABAergic terminals, and reduced spontaneous and evoked neurotransmitter release in neocortical neurons^9^	Missler et al. (2003)^9^
			Constitutive KO Nrxn1/2/3 (only α-neurexins) showed that they regulate N and P/Q type Ca^2+^ channels^10^	Zhang et al. (2005)^10^
		Conditional KO^11,12,13,14,15^	Conditional ablation of neurexins from parvalbumin-positive interneurons in the prefrontal cortex caused a loss of synapses without any effect on action-potential induced Ca^2+^ influx, or synaptic strength. Conditional ablation of neurexins from somatostatin-positive interneurons in the same brain region impaired action-potential induced Ca^2+^ influx and decreased synaptic strength without changes in number of synapses^11^	Chen et al. (2017)^11^
			Conditional KO of Nrxn1/2/3 (only β-neurexins) showed that they regulate postsynaptic endocannabinoid signaling through suppressing CB1 receptor agonist 2AG synthesis in hippocampal synapses. Despite the low expression of β-neurexins, cKO of Nrxn1/2/3 (only β-neurexins) lead to a two-fold decrease in EPSC amplitude, release probability, and action potential induced Ca^2+^ influx^12^	Anderson et al. (2015)^12^
			Conditional KO of Nrxn1/2/3 (all α-and β-neurexins) at the Calyx of Held synapses revealed neurexins regulate neurotransmission through coupling of presynaptic calcium channels to release sites^13^	Luo et al. (2020)^13^
			Conditional KO of Nrxn1/2/3 (all α-and β-neurexins) from four central synapses: excitatory Calyx of Held synapses in the brainstem, excitatory and inhibitory synapses in pyramidal neurons in the CA1 region of the hippocampus, and inhibitory basket cell synapses in the cerebellum, showed that neurexins universally regulate presynaptic GABA_B_-receptor signaling, albeit at different magnitude between synapses^14^	Luo et al. (2021)^14^
			Conditional KO of Nrxn 1/2/3 (all α-and β-neurexins) in DA neurons, showed (1) region-specific increase in GABA release from DA terminals (increase in the ventral but not in the dorsal striatum) and (2) slower DA reuptake along with decreased and increased densities of DAT and VMAT2, respectively^15^	Ducrot et al. (2021)^15^

KO, knockout; KI, knockin; mEPSCs, miniature excitatory postsynaptic currents; mIPSCS, miniature inhibitory postsynaptic currents; NMDARs, N-Methyl-D-aspartate receptors; AMPARs, α-amino-3-hydroxy-5-methyl-4-isoxazole propionic acid receptors; EPSCs, excitatory postsynaptic currents; sEPSCs, spontaneous excitatory postsynaptic currents; sIPSCs, spontaneous inhibitory postsynaptic currents; LTP, long-term potentiation; GABA, γ-aminobutyric acid; DA, dopamine; DAT, dopamine transporter; VMAT2, vesicular monoamine transporter-2. Superscript numbers listed under “brain region examined”; “Genetic approach/manipulation” and “Main findings in synaptic function” corresponds to the respective study listed under “References.”

### 3.2. Nrxn1αβ and Nrxn3αβ

Individual neurexin gene deletions have further attested to the diverse and non-canonical role of neurexins at synapses. Homozygous deletion of Nrxn1 (only α-neurexins) significantly reduced spontaneous release in excitatory glutamatergic synapses without any effect on inhibitory transmission in pyramidal neurons in the CA1 region of the hippocampus (Etherton et al., 2009). Consistent with pan-Nrxn cKO approach, conditional deletion of *Nrxn3* gene in two different regions (CA1 region of the hippocampus and in the olfactory bulb) resulted in two distinct phenotypes (Aoto et al., 2015). Conditional deletion of *Nrxn3* gene in the CA1 region of the hippocampus decreased AMPA receptors (AMPARs)-mediated excitatory response and blockade of NMDARs-mediated LTP. In olfactory bulb synapses however, conditional deletion of *Nrxn3* gene decreased GABAergic mediated inhibitory responses (Aoto et al., 2015). Enforcement to constitutively include splice site 4 (SS4+) (a splice site shared by all neurexin transcripts), in Nrxn3 caused a decrease in AMPARs mediated synaptic responses in hippocampal synapses (Aoto et al., 2013). Inclusion of SS4+ in Nrxn1 increased NMDARs-mediated EPSCs without affecting AMPARs-mediated EPSCs in the CA1-subiculum synapses in the hippocampus. On the other hand, Nrxn3^SS4+^ suppressed AMPARs-mediated synaptic responses without any effect on NMDARs function in the same synapses (Dai et al., 2019). Taken together these studies suggest that Nrxn1 and Nrxn3 have distinct regulatory roles that are synapse specific (Table 1).

### 3.3. Nrxn2

Since the discovery of the neurexin genes, an increasing number of findings suggest that neurexin-2 gene is distinct from neurexin-1 and neurexin-3 genes. Phylogenetic analysis (Reissner et al., 2013) suggest that neurexin-2 gene diverged from a common progenitor of neurexin-1 and neurexin-3 genes indicating that neurexin-1 and neurexin-3 genes are more closely related to each other than to neurexin-2 gene. *NRXN1* and *NRXN3* genes are unusually long genes (>1 Mbp) while *NRXN2* gene is only 0.117 Mbp (Rowen et al., 2002; Tabuchi and Südhof, 2002). The smaller length of *NRXN2* gene can underlie its unique expression pattern in the developing human cerebral cortex that is distinct from *NRXN1* and *NRXN3*. A study that analyzed human cortical tissue by qRT-PCR at 8–12 postconceptional weeks showed that mRNA levels of *NRXN2* were higher than those of *NRXN1* and *NRXN3* during early stages of brain development (Harkin et al., 2017). The same study showed that NRXN2 localized with markers of axon growth and presynaptic terminals in proliferative layers of the developing cortex indicating a role for NRXN2 in early cortical synaptogenesis (Harkin et al., 2017). Furthermore, mouse studies have shown that *Nrxn1* and *Nrxn3* genes have six different homologous splice sites producing thousands of splice variants while *Nrxn2* gene lacks the sixth splice site (Treutlein et al., 2014). A study showed that inclusion or exclusion of SS4 in Nrxn1 and Nrxn3 regulated AMPARs and NMDARs, whereas inclusion or exclusion of SS4 in Nrxn2 had no effect on AMPARs and NMDARs (Dai et al., 2019). Overall, these studies demonstrate that neurexin-2 gene is distinct from neurexin-1 and neurexin-3 genes across different mammalian species.

Until very recently, only three published studies employed genetic mouse models with constitutive KO to study Nrxn2 (Dachtler et al., 2014; Born et al., 2015; Dachtler et al., 2015). From these studies, it was shown that Nrxn2α and Nrxn2αβ KO mice exhibited reduced spontaneous transmitter release at excitatory synapses in the neocortex (Born et al., 2015). Additionally, both KOs exhibited altered facilitation and NMDAR function due to a reduction in NMDAR-dependent decay time and responses in excitatory synapses (Born et al., 2015). Inhibitory transmission and synapse densities and ultrastructure remained unchanged in both Nrxn2α and Nrxn2αβ KO (Born et al., 2015). Nrxn2α KO mice had significant decreases in mRNA of genes encoding for proteins involved in both excitatory and inhibitory transmission as well as in Munc18-1 in the hippocampus (Dachtler et al., 2015). More recently, (Pervolaraki et al., 2019) used diffusion tensor MRI in optically cleared brain tissue from Nrxn2α KO mice and discovered altered microstructure and structural connectivity patterns in the cortex, hippocampus and amygdala, that are brain regions implicated in ASD (Pervolaraki et al., 2019).

While these studies are informative, they do not address the synapse specificity required to effectively study Nrxn2. Moreover, the Nrxn2αβ mouse model by Born et al. (Born et al., 2015) was never able to show complete deletion of *Nrxn2* gene, and the mouse generated was later abandoned because there was no clear evidence that *Nrxn2* gene was deleted (Lin et al., 2023). The first study to comprehensively examine Nrxn2 employed constitutive, conditional and neuron-specific deletion strategies. Neuron-specific deletion of Nrxn2 was employed to account for glial contributions to synaptic phenotypes. Lin et al. discovered (1) the constitutive deletion of *Nrxn2* gene increased hippocampal CA3 to CA1 synaptic connections, (2) neuron-specific deletion of *Nrxn2* gene increased CA3 to CA1 synaptic connectivity, release probability, and excitatory synapse density in the CA1 region, (3) conditional deletion of *Nrxn2* gene enhanced CA3 to CA1 synaptic connections (Lin et al., 2023). Taken together, this comprehensive study suggests that Nrxn2 regulates synaptic connectivity by restricting excitatory synaptic connections in hippocampal circuits. These exciting findings indicate that functions of Nrxn2 are distinct from those of Nrxn1 and Nrxn3 wherein Nrxn1 and Nrxn3 promote synaptic function and Nrxn2 restricts synaptic function in the hippocampus.

Findings from Lin et al. (2023) showed that Nrxn2 regulates synaptic connectivity by inhibiting excitatory synaptic connections in hippocampal circuits. Whether excitatory or inhibitory neuron specific Nrxn2 regulates this restrictive function on excitatory synapses remains unclear. A recent study involved deletion of *Nrxn2* gene under the *Emx1Cre* promoter (Haile et al., 2022) which is predominately expressed in excitatory neurons of neocortex and hippocampus (Boncinelli et al., 1995; Gorski et al., 2002). *Emx1Cre* driven deletion of *Nrxn2* gene increased network activity in hippocampal circuities as measured by increased sEPSC and sIPSC frequencies independent of changes in neurotransmitter release probability or changes in AMPARs and NMDARs contributions (Haile et al., 2022). There was an increase in number of immature spines with no changes in mature spines or dendritic branching in the CA1 region of the hippocampus or cellular layers in the cortex. Although previous studies have shown a canonical regulatory role of neurexins on presynaptic GABA_B_ receptors (Luo et al., 2021), *Emx1Cre* driven deletion of *Nrxn2* gene showed that Nrxn2 might not share this canonical role (Haile et al., 2022). Most intriguingly, the group observed spontaneously reoccurring electrographic and behavioral seizures which have previously never been demonstrated in any other mutant mouse models for neurexins to date (Haile et al., 2022).

## 4. Behavioral abnormalities in *Neurexin* mutant mouse models relevant to ASD

### 4.1. Nrxn1αβ, Nrxn2αβ, and Nrxn3αβ

The effects of a conditional deletion of all three neurexin genes within specific circuits in the hippocampus or striatum on behavioral regulation has been investigated to some extent. Conditional deletion of Nrxn1/2/3 (only β-neurexins) in the hippocampal CA1 region impaired contextual memory without any impairments in cue-dependent memory (Anderson et al., 2015). In another study, the pan-Nrxn cKO approach in DA neurons reduced amphetamine-induced locomotor activity without affecting other striatal-dependent behaviors such as motor coordination or sucrose preference. This investigation suggests that DAT-IRES-Cre driven deletion of neurexin genes can impair DAT function (expressed on DA neurons) leading to altered behavioral responses to DA-acting drugs (Ducrot et al., 2021).

### 4.2. Nrxn1 and Nrxn3 KO mouse models

Constitutive deletion of Nrxn1 (only α-neurexins) induced ASD-related typical (social behavior, communication, repetitive behavioral patterns) and associated (anxiety, cognition) behavioral symptoms (Etherton et al., 2009; Grayton et al., 2013; Dachtler et al., 2015; Armstrong et al., 2020). Certain ASD-behavioral deficits (social approach, anxiety, locomotor activity) were reported from Nrxn1α KO mice on a pure genetic background (Grayton et al., 2013; Armstrong et al., 2020), but not on a mixed background (Etherton et al., 2009; Dachtler et al., 2015), suggesting the importance of transferring the allele to a pure genetic background to understand its role in disease pathophysiology. Furthermore, a mixed genetic background can influence regions outside of the targeted locus, thus confounding behavioral assessment (Reichelt et al., 2012). Conditional deletion approaches have also been employed in studying the role for Nrxn1 in behavioral regulation. Conditional deletion of Nrxn1 (only β-neurexins) from excitatory forebrain neurons in mice induced social approach deficits and repetitive behavioral patterns (Rabaneda et al., 2014). Constitutive absence of Nrxn1 (SS4-) transcripts in the hippocampal CA1 region induced spatial and contextual memory deficits (Dai et al., 2019). Despite the presence of *NRXN3* mutations in ASD, there are no comprehensive investigations that have been undertaken in constitutive *Nrxn3* KO mice. To date, a conditional approach has been employed in which deletion of *Nrxn3* gene from the inhibitory granule cells of the olfactory bulb increased the latency to find buried food confirming the importance of Nrxn3 in inhibitory synaptic transmission in olfactory bulb (Aoto et al., 2015). Hence, a significant amount of effort needs to be directed in investigating the role of Nrxn3 in ASD-related typical and associated symptoms at the preclinical level.

### 4.3. Nrxn2 KO mouse models

Constitutive and conditional KO mouse models for *Nrxn2* gene have produced behavioral manifestations relevant to typical and associated symptoms of ASD, identifying *NRNX2* as a potential candidate gene in the etiology of ASD.

#### 4.3.1. Constitutive Nrxn2 KO mouse models

The constitutive deletion of Nrxn2 (only α-neurexins) induced aberrations in various measures of social behavior including social approach, social novelty, and social investigation. The homozygous deletion of Nrxn2 (only α-neurexins) in mice induced deficits in social approach behavior without any impairments in preference for social novelty (Dachtler et al., 2014). However, in the study by Born et al. (2015), these social approach deficits were only observed in female, but not male, Nrxn2α KO mice, indicating plausible sex differences in social approach behavior in Nrxn2α KO mice (Born et al., 2015). Furthermore, in the same study, female Nrxn2α KO mice engaged in less sniffing time with a conspecific mouse, indicating reduced social investigative behavior (Born et al., 2015). Similar results were produced from the heterozygous deletion of Nrxn2 (only α-neurexins) that yielded mice with deficits in social approach behavior and impaired preference for social novelty (Dachtler et al., 2015). In addition to social behaviors, the impact of constitutive *Nrxn2* gene deletion on other ASD-related behaviors including repetitive behavioral patterns was also investigated, albeit to a much lesser extent. Female, but not male, Nrxn2α KO mice spent more time in self-grooming without any changes in frequency of grooming bouts suggesting sex-dependent role for Nrxn2 in behavioral repetition (Born et al., 2015).

The impact of a constitutive deletion of *Nrxn2* gene on associated symptoms of ASD including anxiety behavior and cognitive behaviors has also been investigated. Nrxn2α KO mice exhibited anxiogenic behavior in paradigms that exploit the natural aversion of mice to exposed areas including open field, elevated plus maze, and light/dark box tests (Dachtler et al., 2014; Born et al., 2015). Interestingly, heterozygous deletion of Nrxn2 (only α-neurexins) did not induce anxiogenic behavior in mice in the open field or elevated plus maze test (Dachtler et al., 2015). Finally, *Nrxn2* gene deletion did not have any effect on cognitive behaviors including information processing and various types of memory function (spatial and working; Dachtler et al., 2014; Born et al., 2015; Dachtler et al., 2015).

#### 4.3.2. Conditional Nrxn2 KO mouse models

The rationale for generating cKO mouse models is that neurexins can have distinct fundamental roles in different synapses highlighting their diverse role in synaptic transmission, circuit function and potentially, behavioral regulation (Chen et al., 2017). In one study, conditional deletion of *Nrxn2* gene in Emx1*-*positive cells in the cortex and hippocampus induced social impairments and repetitive behavioral patterns, which is similar to findings obtained from constitutive KO mouse models (Haile et al., 2022). In this study, female, but not male, *Nrxn2* KO mice exhibited social approach deficits as evidenced by lack of preference between peer and novel object in the 3-chamber social approach task (Haile et al., 2022). Furthermore, male *Nrxn2* KO mice exhibited increased preference for non-social cues and a decreased peer/object ratio was observed in both male and female *Nrxn2* KO mice indicating social approach deficits in both sexes (Haile et al., 2022). In the domain of repetitive behaviors, male, but not female, *Nrxn2* KO mice exhibited increased nestlet shredding behavior without any impairments in other measures of repetitive behaviors including self-grooming, jumping and marbles buried; however, these findings were not accompanied by ASD-related associated symptoms including anxiety-like behavior and working memory deficits (Haile et al., 2022). Another study analyzed hippocampal-specific deletion of *Nrxn2* gene on day 24 postpartum (dpp; Lin et al., 2023). This group did not observe alterations in open field test, fear conditioning or passive avoidance tests, but a small impairment in the rotarod test was observed in *Nrxn2* cKO mice (Lin et al., 2023). Interestingly, the *Nrxn2* cKO mice were able to acquire the location of hidden platform in the water T-maze test as quickly as the WT littermates but exhibited reversal learning deficits suggesting a role for hippocampal Nrxn2 in cognitive flexibility (Lin et al., 2023). Thus, *Nrxn2* gene deletion induced specific, rather than generalized, effects on cognitive behaviors. Considering that cognitive inflexibility has been documented in ASD (South et al., 2012; Lecavalier et al., 2020), *Nrxn2* gene deletion from the hippocampus can recapitulate some, but not all, of cognitive deficits of ASD. Overall, these findings begin to identify a role for the deletion of *Nrxn2* gene in the manifestation of behavioral aberrations that are typical and associated with ASD pathophysiology.

## 5. Future directions

The current work highlights neurexin mutations in ASD and how these mutations can contribute to impairments in synaptic transmission, leading to E/I imbalances and behavioral dysregulation at the circuit level. While the types of neurexin mutations in ASD patients have been deciphered extensively, the outcome of those genetic mutations on protein expression of neurexins in different brain regions has not been thoroughly investigated. These investigations are important in elucidating novel circuit mechanisms though which altered neurexin expression can induce behavioral deficits in ASD patients.

An extensive effort has been undertaken in elucidating the delineation of the heterogenous function of neurexin genes in synaptic physiology. Studies focused on synaptic density, morphology, and transmission have demonstrated that Nrxn2 in hippocampal circuits has a unique and completely opposite role from the currently understood functions of Nrxn1 and Nrxn3. Moreover, these recent findings have transformed previous understandings of neurexins that these SAMs are redundant or have a canonical role. While this presents an exciting prospective avenue of research there are still several gaps in our understanding of the behavioral implications of neurexin deletion in specific circuits in mouse models. Despite the prevalence of *NRXN3* mutations in ASD patients (Vaags et al., 2012; Wang et al., 2018; Yuan et al., 2018), there is very little data regarding the effects of *Nrxn3* mutations in animal models on behavioral abnormalities reminiscent of ASD. Animal models for *Nrxn1* and *Nrxn2* genes provide a strong argument for role of disruption of neurexin function in social behavioral and communication deficits that represent the classical symptoms of ASD. More importantly, *NRXN1* mutations have been reported in children with speech and language impairments (Brignell et al., 2018). Such clinical findings demonstrate the translational validity of these preclinical models in unravelling novel genetic mechanisms underlying socio-communications impairments in ASD. On the other hand, role of neurexins in repetitive behaviors appears to be more limited with studies reporting significant effect of neurexin deletion on some, but not all, of measures of repetitive behavior in preclinical models. Additionally, the effects of neurexin deletion on cognitive, motor and anxiety behaviors are also limited. Future studies such as conditional deletion of neurexin genes from specific brain regions such as the amygdala that controls social behavior (Felix-Ortiz and Tye, 2014), anxiety (Duvarci and Pare, 2014) and cognitive functions (McGaugh, 2004) would be warranted to ascertain a role for neurexins in ASD-related behaviors. This would be important in the case of Nrxn1 and Nrxn3 since there are few studies (or none) that investigated the effects of conditional deletion of either *Nrxn1* or *Nrxn3* genes on classical and associated behaviors reminiscent of ASD. In contrast, significant milestones have been achieved in determining the effects of conditional deletion of *Nrxn2* gene on ASD-related behaviors (Haile et al., 2022; Lin et al., 2023). Nevertheless, these shortcomings do not undermine the contribution of neurexins to ASD pathophysiology since one animal model cannot recapitulate all the behavioral symptoms of ASD. Furthermore, the conditional deletion of all three neurexin genes on circuit-specific behaviors in the hippocampus or brain regions relevant to ASD pathophysiology needs to be thoroughly investigated. The mutations in neurexin genes can play an essential role in elucidating specific pathways underlying ASD endophenotypes and can aid in the development of druggable targets at the synapses. Moreover, future studies in studying neurexin-glial interactions, the role of sex in neurexin-dependent behaviors, and their role in genetic mechanisms underlying comorbidities will be crucial for deciphering the role of neurexins in ASD pathophysiology.

## Author contributions

SK, MTH, and LYC: writing and editing. MTH: compiled supplementary data and made table. LYC: supervision. All authors contributed to the article and approved the submitted version.

## Funding

This work was supported by the UCI School of Medicine Startup fund (GF15247 to LYC).

## Conflict of interest

The authors declare that the research was conducted in the absence of any commercial or financial relationships that could be construed as a potential conflict of interest.

## Publisher’s note

All claims expressed in this article are solely those of the authors and do not necessarily represent those of their affiliated organizations, or those of the publisher, the editors and the reviewers. Any product that may be evaluated in this article, or claim that may be made by its manufacturer, is not guaranteed or endorsed by the publisher.
